# Achieving optimal orthodontic results with hybrid strategies: leveraging the synergy of aligners and fixed appliances

**DOI:** 10.1590/2177-6709.30.4.e25spe4

**Published:** 2025-11-07

**Authors:** Renato Parsekian MARTINS

**Affiliations:** 1 MSc, PhD, and Postdoctoral Fellowship in Orthodontics at Universidade Estadual Paulista, School of Dentistry at Araraquara (São Paulo, Brazil).; 2 Post Doc at the Orthodontic Department at Baylor College of Dentistry (now Texas A&M School of Dentistry) (Dallas/TX, USA).; 3 Associate Editor of the Dental Press Journal of Orthodontics and Revista Clínica de Ortodontia Dental Press.; Revista Clínica de Ortodontia Dental Press, Brazil; 4 Clinical Advisory Council of the Journal of Clinical Orthodontics.

**Keywords:** Orthodontics, Clear Aligner Appliance, Orthodontic Wires, Ortodontia, Alinhadores ortodônticos, Fios ortodônticos

## Abstract

**Introduction:**

Despite their aesthetic appeal and rising popularity, clear aligners face significant limitations in performing complex orthodontic movements such as anterior extrusion, severe rotations, and efficient molar distalization. These biomechanical challenges often result in longer treatment durations and reduced predictability.

**Objective:**

This article explores the hybrid treatment approach, which combines the biomechanical efficiency of fixed appliances in the initial phase with the aesthetic and hygienic advantages of aligners in the finishing phase. Fixed appliances are employed to manage movements that aligners struggle to control reliably, including root torque, vertical extrusion with proclination, and large rotational corrections. Once these key adjustments are made, aligners are introduced to complete treatment with greater comfort and patient satisfaction.

**Conclusion:**

By strategically integrating both systems, hybrid therapy enhances clinical efficiency, reduces total treatment time, and delivers personalized care aligned with modern patient expectations.

## RESUMO

**Introdução:**

Apesar de seu apelo estético e crescente popularidade, os alinhadores enfrentam limitações significativas na realização de movimentos ortodônticos complexos, como extrusão anterior, rotações severas e distalização eficiente dos molares. Esses desafios biomecânicos frequentemente resultam em maior duração do tratamento e menor previsibilidade.

**Objetivo:**

O presente artigo explora a abordagem híbrida, que combina a eficiência biomecânica dos aparelhos fixos na fase inicial com as vantagens estéticas e de higiene dos alinhadores na fase de finalização. Os aparelhos fixos são utilizados para controlar movimentos que os alinhadores não executam com previsibilidade, como controle radicular, extrusão com proclinação e grandes rotações. Após essas correções, os alinhadores são empregados para finalizar o tratamento com maior conforto e aceitação por parte do paciente.

**Conclusão:**

Ao integrar estrategicamente ambos os sistemas, a terapia híbrida melhora a eficiência clínica, reduz o tempo total de tratamento e proporciona um atendimento personalizado, alinhado às expectativas do paciente moderno.

## INTRODUCTION

The increasing adoption of orthodontic aligners reflects a significant shift in patient preferences, driven by enhanced awareness of aesthetic dental solutions, advancements in digital orthodontic technologies, and a growing demand for personalized treatment options. Aligners offer a discreet and comfortable alternative to traditional fixed appliances, positioning themselves as an appealing choice for many patients. However, despite their rising popularity, aligners have not consistently achieved the outcomes originally projected in treatment software.^1^

Specifically, aligners struggle with certain tooth movements, such as root control,^2^ ^-^ ^6^ pure vertical movements^2^ ^,^ ^6^ ^,^ ^7^ and complex antagonistic movements like incisor uprighting combined with intrusion or incisor proclination with extrusion. Additionally, while aligners can correct severe rotation or distalize molars,^2^ ^,^ ^5^ their efficiency in executing these movements is often lacking. Aligners typically require significantly more time compared to fixed appliances or when used with supplementary mechanics, making them less effective in achieving timely results for these complex adjustments.

Pure extrusion or extrusion with proclination remains particularly challenging for aligners^2^ ^,^ ^8^ ^,^ ^9^ due to their reliance on the elasticity of the plastic and patient compliance, rather than precise load system found in fixed appliances. In addition to vertical challenges, severe rotations pose significant difficulties for aligners.^2^ ^,^ ^5^ ^,^ ^6^ ^,^ ^8^ ^-^ ^10^ Although techniques such as round-tripping, overcorrection, and refinements can increase the reliability of rotational corrections, fixed appliances excel due to their ability to exert the continuous forces and the couples necessary for efficient tooth derotation. Using brackets and wires before aligners to correct severe rotations might establish a favorable starting point for subsequent aligner therapy.

Distalization, particularly in achieving anterior-posterior (AP) corrections, highlights these challenges. While aligners can facilitate distalization of maxillary molars by up to 1.5 mm,^2^ ^,^ ^5^ the prolonged time required for such movements can pose issues with patient compliance and treatment efficiency. Fixed appliances or supplemental mechanics, like carriere-type distalizers associated with intermaxillary elastics or skeletal anchorage, can achieve equivalent results much faster, offering a more time-efficient solution.

The hybrid treatment approach leverages the strengths of both fixed appliances and aligners. By starting with fixed appliances to address these challenging movements, practitioners can capitalize on their capabilities for specific adjustments. Once foundational corrections are made, transitioning to aligners offers advantages in aesthetics, comfort, oral hygiene, and patient satisfaction. This strategy enhances treatment efficiency, reduces overall treatment time, and delivers high-quality results tailored to individual needs.

In conclusion, the strategic use of fixed appliances during the initial phase of treatment facilitates complex tooth movements that are difficult or less predictable with aligners alone. Once these corrections are achieved, aligners can take over to fine-tune and finish the treatment, offering a comprehensive solution that addresses both clinical needs and patient preferences. This synergy exemplifies the success of hybrid orthodontics in modern practice. This article aims to explore the hybrid treatment approach that combines the strengths of both aligners and fixed appliances to optimize orthodontic outcomes.

## CHALLENGES IN EXTRUSION OF ANTERIOR TEETH

Extrusion is recognized as the most challenging movement to control with aligners,^2^ ^,^ ^6^ with accuracy rates ranging from 29% to 54%.^2^ ^,^ ^8^ ^,^ ^9^ Research indicates that bite closure when using aligners primarily results from changes in incisor positions, accompanied by minimal alterations in molar positioning.^11^ ^,^ ^12^ Furthermore, evaluations of the literature reveal that most of these changes stem from retroclination rather than pure extrusion.^12^ It is also important to note that tipping is likely the most reliably achieved movement with aligners.^6^ ^,^ ^13^ Consequently, it can be concluded that achieving pure extrusion-especially when combined with proclination of the incisors, which represents an antagonistic movement-would be even more difficult, if not nearly impossible.

**Figure 1:**
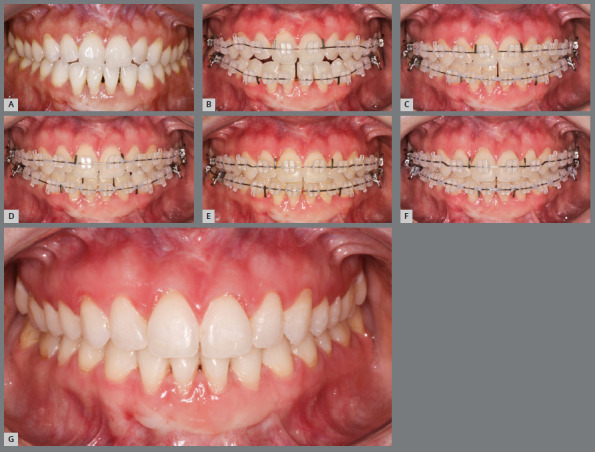
Open bite correction using hybrid orthodontic approach. **A)** Patient presenting with an anterior open bite requiring extrusion of maxillary incisors without lingual tipping. **B)** Biomechanics aimed at rapid bite closure: 0.022-in ceramic brackets (Roth prescription; Orthometric, Marília, Brazil) bonded and 0.014-in superelastic NiTi wire inserted. Rectangular NiTi wire segments were spot-welded to the main archwire, and anterior vertical box elastics (1/4”, 8 oz) were used (K-hooks could be substituted). **C)** Progress one month after bonding. **D)** Progress two months after bonding. **E)** At three months: 0.016 × 0.022-in NiTi archwire with vertical bends placed to allow overcorrection. **F)** Four months post-bonding: fixed appliances removed to transition to aligners. Approximately 4 mm of incisor extrusion achieved in four months; equivalent movement with aligners alone would require ~40 aligners (0.1 mm per aligner) or 20 months, plus overcorrection protocols increasing total to ~60 aligners (~30 months). **G)** Final outcome following completion with aligner therapy.

In situations where retroclination of incisors is not permissible or preferred, especially when combined with incisor extrusion, it is advisable to achieve these movements using fixed appliances before commencing aligner therapy. Anterior extrusive mechanics, utilized effectively along with anterior box elastics,^14^ ^,^ ^15^ can facilitate and support the necessary vertical movement in a timely manner. Once the bite has closed effectively, aligners can then be employed to address the remaining aspects of the malocclusion (Fig 1).

## CHALLENGES IN CORRECTING SEVERE ROTATIONS

**Figure 2:**
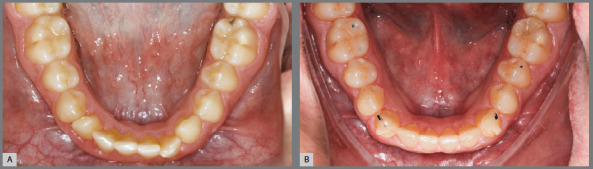
Patient outcomes for severe rotation correction. A) Patient presenting a severe rotation in the lower right canine. B) Rotation corrected exclusively with aligners.

**Figure 3:**
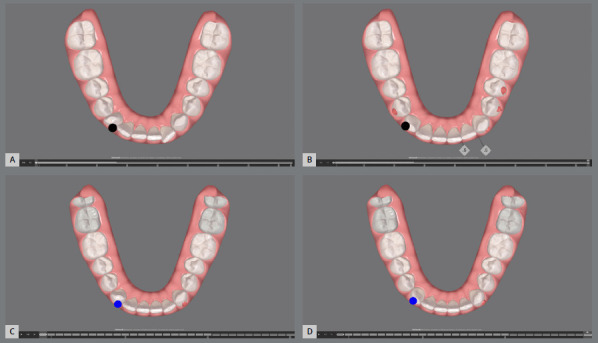
Treatment planning and progress for the patient in Figure 2. A) Initial intraoral scan of the patient shown in Figure 2, imported into aligner treatment planning software. (ClinCheck; Align Co., Tempe, AZ, USA ). B) Predicted treatment outcome after 84 aligners. C) Intraoral scan taken after completion of the initial aligner series. D) Refinement treatment plan involving an additional 34 aligners.

The correction of severe rotations is particularly challenging to manage with aligners,^2^ ^,^ ^5^ ^,^ ^6^ ^,^ ^9^ especially in premolars and canines.^2^ ^,^ ^9^ ^,^ ^10^ To improve the reliability of corrections, strategies such as intentional overcorrection,^5^ ^,^ ^16^ ^-^ ^18^ decreasing the velocity of movement^5^ and/or refinements^13^ ^,^ ^19^ should be utilized. While it is technically possible to achieve significant rotational corrections with aligners, the time required is a critical factor. Excessive treatment time can negatively impact patient compliance, which may, in turn, affect the final treatment outcome. Although achieving rotations exceeding 60 degrees with aligners alone is feasible, (Figs 2 and 3) the prolonged treatment duration required may not be practical or reasonable.

In contrast, fixed appliances typically can correct rotations much more quickly compared to aligners. Fixed appliances’ biomechanical benefits are notable, as they can apply consistent and continuous forces around the clock, allowing for more efficient and effective rotation corrections. While we are not considering the aesthetic advantages of aligners in this context, these properties make brackets highly effective for treating severe rotations. 

**Figure 4:**
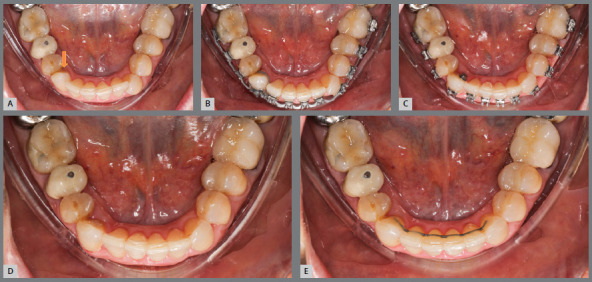
Hybrid treatment overview. A) Patient with a severe rotation in the lower right canine. B) A 0.014-inch superelastic NiTi wire was engaged with 0.018-inch Roth prescription brackets bonded from first molar to first molar (Opal Orthodontics, Sandy, Utah ). Note that chain elastics were used to create a couple in the lower right canine and a moment of a force on the lower left canine. C) Three months after initial bonding, the rotations have been resolved. D) The brackets were removed to transition to aligner therapy. E) Four months later, the case was completed using aligners.

Therefore, in situations where severe rotations are present, it may be advantageous to initially correct them using fixed appliances over a span of 3-4 months. After addressing the severe rotations, transitioning to aligners can be beneficial for completing the remainder of the treatment, taking advantage of their aesthetic appeal and patient comfort (Fig 4).

## EFFECTIVENESS OF ALIGNERS IN DISTALIZATION

Aligners can be effective in achieving anterior-posterior (AP) correction.^2^ ^,^ ^5^ However, the reliability of this correction varies significantly, with accuracy reported between 7% and 88%,^2^ ^,^ ^20^ ^,^ ^21^ Although aligners can distalize maxillary molars by up to 1.5 mm^2^ ^,^ ^5^ with predictability, combining sequential distalization of the maxillary dentition with mesialization of the mandibular dentition can further improve treatment outcomes. To achieve this effectively, additional mechanics should be employed, such as intermaxillary elastics or intramaxillary elastics with skeletal anchorage. However, the key consideration is not only whether aligners will successfully achieve the desired AP correction, but also the duration required for these movements to occur. Understanding this timeline is crucial for orthodontists to plan treatment effectively and manage patient expectations.

**Figure 5:**
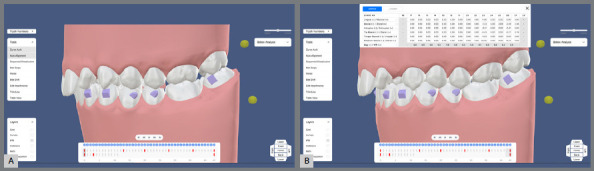
Estimation of sequential distalization of maxillary molars, premolars, and canine. **A)** Initial intraoral scan of the patient imported into aligner treatment planning software. (Archform, Sunnyvale, California, USA). **B)** Estimated outcome using 43 aligners for distalization, with 0.2 mm of movement per aligner and a 50-50 distalization protocol. The distalization process is projected to require approximately two years of treatment.

Mesio-distal movements programmed for each aligner are typically around 0.2 mm per aligner. The distalization protocol often employs a 50-50 movement strategy, where each tooth begins to move only after the preceding tooth has achieved 50% of its targeted movement. For example, the first molar will begin to distalize once the second molar has reached 50% of its intended distalization, and this pattern continues sequentially. This method often requires approximately 45 aligners to achieve a total distalization of 2 to 2.5 mm. Assuming patients change their aligners every 14 days, the distalization process for molars, premolars, and canines is expected to take approximately 22 months (Fig 5).

**Figure 6:**
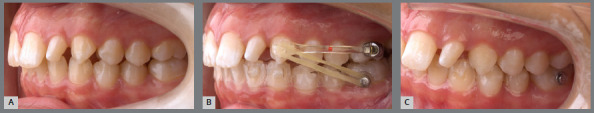
Distalization using hybrid orthodontics (same patient referenced in the estimates made in Figure 5). A) Initial left side intraoral photograph. B) Carriere-style distalizer used in conjunction with mandibular aligners and a ¼” 8 oz. elastic. (Iceram Distalizer; Orthometric, Marilia, Brazil ). C) Class II relationship corrected after 3 months of treatment. (Case treated in collaboration with Dr. Patricia Schneider, São Paulo, Brazil ).

In contrast, alternative methods such as ‘Carriere’-style distalizers or traditional fixed appliance mechanics can achieve equivalent results much faster, typically within 3 to 6 months of treatment. These approaches offer significant advantages in terms of time efficiency, allowing for quicker corrections and potentially improving patient satisfaction with the overall treatment timeline. Once the planned anterior-posterior (AP) corrections are achieved, the patient can transition to aligner treatment, which will require fewer aligners. This strategy not only speeds up the treatment process, but also reduces costs, making it a more efficient option for both practitioners and patients (Fig 6).

## PATIENT ACCEPTANCE AND COMPLIANCE IN CLEAR ALIGNER THERAPY

Patient acceptance is a crucial determinant of the success of orthodontic treatments. It has been demonstrated that clear aligners generally garner higher patient acceptance due to their aesthetic appeal and comfort.^22^ ^-^ ^24^ In the context of hybrid treatments, patient education is pivotal in fostering acceptance. When patients are thoroughly informed about the necessity and benefits of hybrid approaches, their willingness to undergo such treatments increases significantly.^25^ Patients are more inclined to accept hybrid orthodontic approaches when it is explained that the initial use of fixed appliances can expedite challenging movements, thereby enhancing their appreciation for the subsequent transition to aligners, which offer improved aesthetics and comfort. Moreover, this strategic combination not only aligns with patient preferences but also contributes to a reduction in overall treatment time, thereby enhancing both clinical efficiency and patient satisfaction, particularly with the use of ceramic brackets. This understanding can be achieved through comprehensive consultations that elucidate the rationale behind each phase of the treatment, set realistic expectations, and foster trust.

It may not be difficult to convince a patient to use fixed appliances for a few months, especially if they are clear appliances, to facilitate complex movements or significantly decrease the overall duration of orthodontic treatment.

## CONCLUSION

In summary, while aligners provide a more practical and aesthetic approach for patients compared to fixed appliances, they face significant limitations in controlling complex tooth movements such as severe rotations, extrusion of anterior teeth, and achieving timely distalization. Their reliance on patient compliance and the nature of the material often lead to challenges in achieving timely and predictable outcomes. In contrast, fixed appliances, with their ability to apply continuous forces and produce reliable load systems, allow for more efficient and effective corrections, particularly for challenging movements.

The hybrid treatment approach leverages the strengths of both fixed appliances and aligners. By initially utilizing fixed appliances to manage complex movements, orthodontists can establish a solid foundation for treatment. Once difficult movements are simplified and time-consuming issues are resolved, transitioning to aligners allows for the delivery of final adjustments while also enhancing the aesthetic appeal and comfort for patients.

Ultimately, the strategic integration of both treatment modalities not only improves overall treatment outcomes but also aligns with patient preferences for enhanced aesthetics and comfort. This comprehensive approach exemplifies the evolving landscape of orthodontics, where the synergy between traditional mechanics and innovative technology can fulfill the clinical needs and expectations of modern patients.
